# Structured feedback on students’ concept maps: the proverbial path to learning?

**DOI:** 10.1186/s12909-017-0930-3

**Published:** 2017-05-25

**Authors:** Conran Joseph, David Conradsson, Lena Nilsson Wikmar, Michael Rowe

**Affiliations:** 1 Department of Physiotherapy, University of the Western Cape, Cape Town, South Africa; 2 Department of Neurobiology, Care Sciences and Society, Karolinska Institutet, Stockholm, Sweden; 3 Karolinska University Hospital, Functional Area Occupational Therapy & Physiotherapy, Allied Health Professionals Function Stockholm, Stockholm, Sweden; 4 Academic Primary Healthcare Centre, Stockholm County Council, Stockholm, Sweden

**Keywords:** Concept mapping, Structured feedback, Conceptual knowledge, Meaningful learning

## Abstract

**Background:**

Good conceptual knowledge is an essential requirement for health professions students, in that they are required to apply concepts learned in the classroom to a variety of different contexts. However, the use of traditional methods of assessment limits the educator’s ability to correct students’ conceptual knowledge prior to altering the educational context. Concept mapping (CM) is an educational tool for evaluating conceptual knowledge, but little is known about its use in facilitating the development of richer knowledge frameworks. In addition, structured feedback has the potential to develop good conceptual knowledge. The purpose of this study was to use Kinchin’s criteria to assess the impact of structured feedback on the graphical complexity of CM’s by observing the development of richer knowledge frameworks.

**Methods:**

Fifty-eight physiotherapy students created CM’s targeting the integration of two knowledge domains within a case-based teaching paradigm. Each student received one round of structured feedback that addressed *correction, reinforcement, forensic diagnosis, benchmarking,* and *longitudinal development* on their CM’s prior to the final submission. The concept maps were categorized according to Kinchin’s criteria as either *Spoke*, *Chain* or *Net* representations, and then evaluated against defined traits of meaningful learning.

**Results:**

The inter-rater reliability of categorizing CM’s was good. Pre-feedback CM’s were predominantly *Chain* structures (57%), with *Net* structures appearing least often. There was a significant reduction of the basic *Spoke-* structured CMs (*P* = 0.002) and a significant increase of *Net*-structured maps (*P* < 0.001) at the final evaluation (post-feedback). Changes in structural complexity of CMs appeared to be indicative of broader knowledge frameworks as assessed against the meaningful learning traits.

**Conclusions:**

Feedback on CM’s seemed to have contributed towards improving conceptual knowledge and correcting naive conceptions of related knowledge. Educators in medical education could therefore consider using CM’s to target individual student development.

## Background

The goal of teaching is to help students acquire the requisite knowledge and skills that are necessary for clinical practice [1]. Health professions education has traditionally emphasized the development of procedural knowledge whereby students learn how to perform diagnostic and curative techniques [2]. However, teaching should be a process of creating learning activities that allow students to integrate knowledge within different contexts, rather than presenting it as a decontextualised set of abstractions [3]. There is therefore a trend towards focusing students’ attention on the interpretation of concepts and the relationships between them, which facilitates the development of procedural skills – an important attribute for success in the medical field [2, 4].

Although similar treatments are often applied to certain health diagnoses, standardized approaches are not feasible due to the uniqueness of each patient profile. Good conceptual knowledge enables more flexible problem solving because those who understand the rationale behind procedures are more likely to reason their way through novel cases, taking into account the unique profile of each case [4, 5]. Conceptual knowledge is therefore especially important in health professions education because students must not only transfer knowledge and skills from the classroom to clinical practice but also between clinical contexts [6].

Traditionally, conceptual knowledge is assessed using multiple-choice or true and false questions, which do not always provide insight into how students link concepts together and what meaning they ascribe to those relationships [7]. One option for accessing students’ conceptual understanding is to use concept mapping (CM) [8]. Concept mapping is a visual model of relationships between concepts, using explanatory phrases (called propositions) to link two or more concepts together in meaningful ways. Authors of CMs use concepts and propositions to provide two-dimensional sketches of their understanding of a particular knowledge framework. By providing a visual representation of the relationship between concepts, CMs help students organize and structure their thoughts. This in turn allows educators to detect gaps and misconceptions in students’ understanding [9], which can then be addressed with feedback.

Formative feedback primarily aims to reinforce correctness, but can also be used to question erroneous conceptions and proposing new ideas. In order for feedback to be effective it should be timeously provided on multiple occasions with an emphasis on information sharing rather than judgment [10, 11]. However, what remains poorly understood is whether attempts to improve conceptual knowledge in the classroom via feedback leads to improved patient management in the clinical context.

Although CM has been found to foster a critical bridge between theory and practice [6], little is known about the methods used to evaluate CM’s, with both quantitative and qualitative assessments being proposed [12]. The quantitative scoring of CM’s by counting the number of concepts and links between concepts is used in summative evaluation, whereas the qualitative categorization of CM’s is by evaluation of the graphical representation which allows educators to determine changes in students’ thinking [13]. A qualitative evaluation of CM’s based specifically on Kinchin’s criteria [14] could therefore be used to measure changes in patterns of thinking over time [15].

The International Classification of Functioning, Disability and Health (ICF) is an example of a complex knowledge framework used across all health disciplines to understand health and disability [16]. With the lack of appropriate tools to assess conceptual understanding of the ICF in the classroom context, educators may only observe and reflect upon the knowledge gaps of students when they enter the clinical setting. Addressing problems in conceptual knowledge at this late stage could be challenging since students might not be as ‘learning-ready and flexible’ as they were when these ideas were first presented in the classroom [14]. We introduced the use of CM’s as a teaching and evaluation tool to determine if we could detect changes in students’ conceptual knowledge after structured, formative feedback. Specifically, this study set out to answer the following questions:

I. What is the inter-observer reliability of the qualitative categorization of CM’s - based on Kinchin’s criteria - before and after feedback?
II. What is the effect of formative feedback on the graphical complexity of knowledge structures?
III. Do changes in graphical complexity following feedback typify patterns of learning?

Given the prerequisite of good –excellent inter-observer reliability, we hypothesized that individualized, structured feedback would result in significant changes in graphical structure, in that CMs would become more complex. We also assumed that more complex CMs represented deeper patterns of learning [15].

## Method

### Research design

A pretest-posttest quasi-experimental design was used to qualitatively categorize CM’s according to Kinchin’s criteria prior to, and after, educator’s feedback on the initial CM’s. This design, similar to randomized trials, attempts to demonstrate causality between an intervention and the outcome; in this case the effect of structured educator feedback on the complexity of a concept map as a proxy for student learning.

### Participants

This study was conducted during a 7-week course in an undergraduate physiotherapy program. All registered undergraduate physiotherapy students in the second year of a university physiotherapy department in South Africa were included in the study (*n* = 58) and gave consent for their CM’s to be included. This study received ethics clearance from the Senate Research Committee of the University of the Western Cape.

### Learning environment and procedure

The course included the integration of the ICF within realistic case studies that aimed to enable educators to better assess and improve students’ conceptual understanding of the ICF. Students first attended two lectures on the relevant application of the ICF in the clinical context. They were then asked to apply the ICF to a case study that incorporated multiple health conditions. Students were then shown how to install and use the Cmap tools software (IHMC CmapTools, Florida, USA) [17], a freely available and widely used application for developing CM’s. During the information session with students, we (educator and students) collaboratively drew a CM using two related knowledge frameworks, whereby the health condition ‘Stroke’ was unpacked within the impairment domain of the ICF. During the construction of the map the educator asked students to identify all concepts related to stroke impairments, which they had to link to either abnormalities in body structure or function according to the ICF. Following this, students created a CM demonstrating their understanding of the meaningful relationships between concepts from both the case study and the ICF. After submission of the draft CM, the first author provided structured and formative feedback. In order to improve the CM’s, students were encouraged to use all available resources in addition to the feedback they received, i.e. lecture notes and credible online material, and to engage in dialogue with the educator or peers when conceptual difficulties were encountered [18]. Finally, students submitted their revised CM’s 2 weeks later.

### Formative feedback

The feedback helped students identify problems with their representations of conceptual relationships, guiding them to deeper reflection based on questioning of their maps [1, 9]. The feedback aimed to address the following aspects of the CM’s: *correction, reinforcement, forensic diagnosis, benchmarking, and longitudinal development* [11]*.* All concepts and their propositions were assessed for accuracy. If incorrect, the conceptual relationship was questioned, guiding students to clarify or rectify the proposed relationship (positive and negative *reinforcement*). Students were also encouraged to use literature to confirm the relationship between concepts in cases where the relationship was not ‘common knowledge’. In cases where novel associations were proposed by students, a second reviewer was asked to *reinforce* and confirm this. The forensic role of the feedback enabled the diagnosis of problems in the work, for example, erroneous assumptions or poorly described concepts were used to bridge the gap between what was understood and the expected level of understanding. The forensic part of feedback on CM required the student to reflect upon whether a consequence of disease was linked to the simplest building block of the ICF taxonomy. This allowed students to create another level in the CM hierarchy, which is consistent with more exhaustive CM’s. Mastering this step placed students at *benchmark*. These four categories were consistently applied to all student-authored concept maps. Since students would be required to apply the concepts of this course in future clinical settings, longitudinal development was promoted by encouraging them to develop new ways of thinking about the relationship between concepts from different knowledge domains [19, 20]. This longitudinal development is not presented in this paper.

### Data collection instrument

**Fig. 1:**
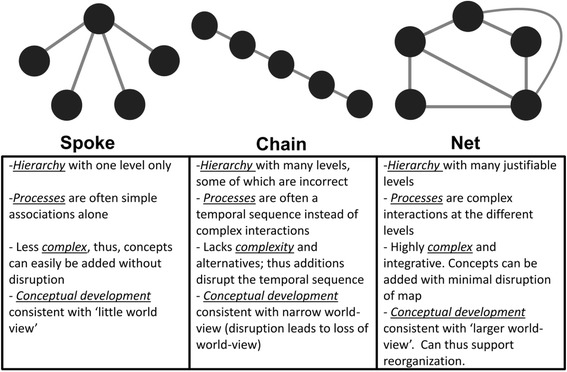
Assessment criteria of different graphical presentations of concept maps

The qualitative analysis in this study was based on Kinchin’s assessment criteria for graphical presentation of maps. These criteria are used to categorize students’ level of conceptual knowledge, since the graphical structure of CMs can be related to patterns of learning [21]. The scheme differentiates maps according to their complexity, resilience in accommodating additions, the establishment of context, and the degree of appreciation of a wider viewpoint, with an end result that categorizes CM’s as either *Spoke, Chain or Net* structures*.* Each CM was initially assessed according to its structural representation and thereafter, feedback was provided to develop general complexity and saturation of the map in order to achieve a level of learning that approached the criterion (*Net* structure). The assessment criteria of each structure are presented in Fig. 1.

### Data analyses

Two raters, the first author and another independent educator, blinded to the assessment of the other, examined the maps according to the three graphical representations of Kinchin’s criteria. The inter-rater reliability was computed using Cohen’s Kappa and the Prevalence-Adjusted Bias-Adjusted Kappa (PABAK) that takes into account chance agreement [22]. The reliability coefficient was interpreted using Landis and Koch [23] criteria for acceptability, which were as follows: values <0 indicate no agreement and 0–0.20 as slight, 0.21–0.40 as fair, 0.41–0.60 as moderate, 0.61–0.80 as substantial, and 0.81–1 as almost perfect agreement. In instances where the raters disagreed, a consensus meeting was held to unanimously allocate the most probable structure of those concept maps. To evaluate the impact of formative educator’s feedback, we compared the difference in proportions of CM’s in the three categories between the first (pretest) and second (posttest) evaluation with Fischer’s Exact test.

Selected pairs of CM’s were analysed for changes in patterns of learning, which are typically characterised by measuring documented change [15]. Hay’s criteria were used to measure the changes in learning as founded in Novak’s concept mapping, which is grounded within Ausubel’s theory of assimilative learning [21, 24]. Furthermore, since Kinchin’s assessment criteria for categorising concept maps are consistent with Novak’s assumptions of changes in learning [17], using Hay’s criteria to measure changes seemed appropriate to assess new information presented in subsequent CMs.

Two raters were responsible for assessing whether meaningful learning had occurred or not. Changes in learning (in this case, the graphical differences between the draft and final CM’s) were measured in order to differentiate between deep, meaningful learning and surface learning. Meaningful learning in this assignment was defined using the following three traits [15]:

1. the learner has prior knowledge (first draft CM without educator feedback) that is relevant to the new learning to be done (second draft CM after tailored feedback was provided). Thus, the second map must show newly learned concepts and original conceptions.
2. that what is to be learned was illustrated in ways that have meaning. The second map must show that the new knowledge has been linked to prior knowledge in a meaningful way, which is evident in the correctness (richness) of linking statements and/or new ways of understanding.
3. that the learner must choose to learn meaningfully, which was evident if the knowledge structure of the second map was a significant improvement of the first (i.e. better organisation and richer meanings between concepts were desirable).

## Results

### Reliability of the evaluation of concept maps

The inter-rater reliability of the CM’s on the first (pre-feedback) and second occasion (post-feedback) was 0.785 (*P* < 0.0001) and 0.713 (*P* < 0.0001) respectively, typifying substantial agreement significantly beyond chance. Since the kappa estimate of reliability is conservative, meaning not accounting for the prevalence of disagreement or bias, we computed the Prevalence-adjusted Bias-adjusted Kappa (PABAK-Ordinal Scale). The PABAK-OS produced agreement coefficients of 0.819 (95%CI: 0.698–0.940; *P* < 0.0001) and 0.767 (95%CI: 0.665–0.869; *P* < 0.0001) between raters on the pre- and post-assessment of CMs, indicating almost excellent and substantial agreement, respectively. These estimates provide evidence for the use of concept mapping as a reliable method of assessing changes in the graphical representation of knowledge in this context.

### Changes in graphical presentation of concept maps pre- and post-educator’s feedback

Overall, a significant difference in the proportions of the three categories of graphical representation was found between pre- and post-feedback assessments (Fischer’s Exact test: *P* < 0.001). As seen in Tables 1 and 2, analysis of the draft (pre-feedback) CM’s revealed that 31% were *Spoke* structures, 57% were categorized as *Chain*, and *Net* structures presented only 12%. The *Spoke*-representative CM’s were significantly reduced to 9% after feedback, whereas the *Chain*-structured maps continued to be the most common (57%). The proportion of complex *Net*-structured maps was significantly higher (*n* = 22; *P* < 0.001) after feedback, compared with the first draft (*n* = 7).

**Table 1:** Graphical categorisation of concept maps using Kinchin’s criteria

	Pre-feedback	Post-feedback	*P* value*
	*N* = 58 (%)	*N* = 58 (%)	
Structure			
Spoke	18 (31)	5 (9)	0.002*
Chain	33 (57)	31 (53)	0.711
Net	7 (12)	22 (38)	0.001*

**Table 2:** Changes in graphical representation of concept maps between pre- and post-feedback submission

Category of map pre-and post-feedback		Number (n)
Spoke (*n* = 18)		
Spoke	Spoke	5
Spoke	Chain	9
Spoke	Net	4
Chain (*n* = 33)		
Chain	Spoke	0
Chain	Chain	22
Chain	Net	11
Net (*n* = 7)		
Net	Spoke	0
Net	Chain	0
Net	Net	7

### Analysis of concept maps for underlying patterns of learning

The total data set consisted of two CM’s for each of the 58 students. The CM’s, pre- and post-educator feedback, of three students are shown below in detail as the clearest examples of observed additions (using the features of the three distinct representations) in the graphical complexity of maps following feedback. The additions and changes were further examined more carefully to assess whether meaningful learning took place [15].

**Fig. 2:**
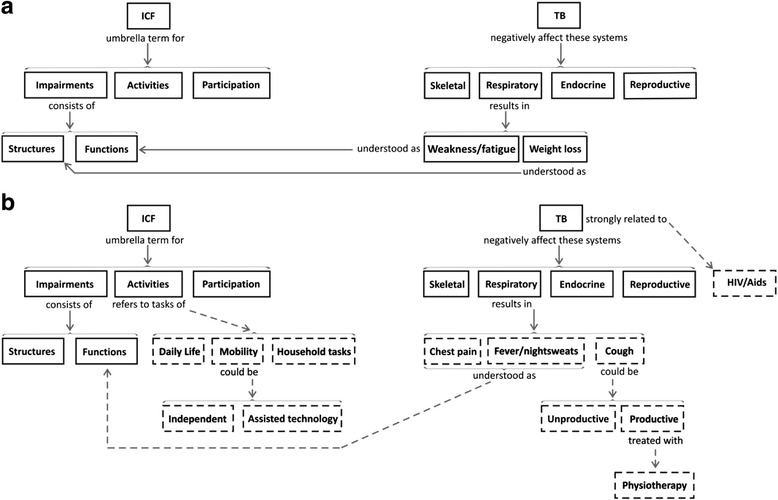
Student 1 with *Spoke* (**a**) and *Chain* (**b**) structures pre- and post- feedback. *Dashed lines* and *boxes* indicate new concepts and linking statements

The initial draft CM of student 1 (see Fig. 2a below) provided the clearest example of a *Spoke* knowledge structure concerning the subject domains. For this knowledge structure, additions to the central concept (i.e. the ICF) could easily be integrated without disrupting the integrity of the map. For example, student 1 failed to present knowledge concerning the importance of the contextual component of the ICF that attempts to understand the impact of the personal profile of the patient and the unique environmental nuances on the experience of living with TB and HIV/AIDS. In the second CM (new concepts inserted in dashed boxes), post-feedback, the student added new knowledge in a meaningful way, resulting in a more comprehensive demonstration of their understanding of the ICF, especially within the “participation” domain. Despite noteworthy additions and improvements, the second map (Fig. 2b) illustrates fundamental gaps in the student’s knowledge about the ICF, for example, the life areas of the “activity” category and the contextual component of the disability model. In terms of learning, this student was able to add new concepts due to the initial very basic CM. However, improvements were not significant enough after feedback to suggest the occurrence of deep and meaningful learning, according to the three traits by Hay [15].

**Fig. 3:**
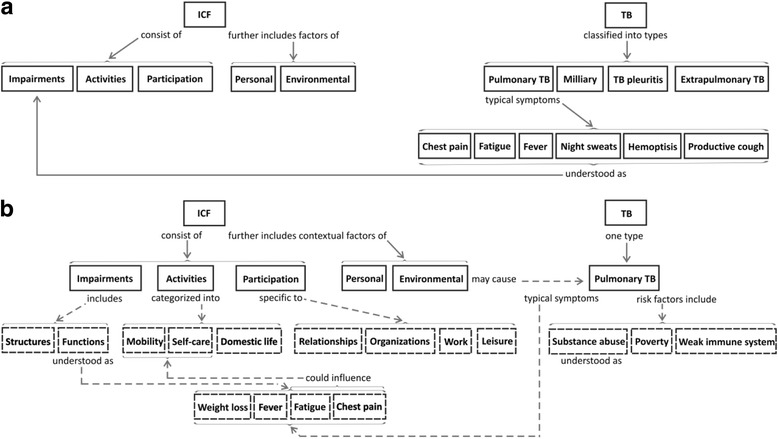
Student 2 with *Spoke* (**a**) and *Net* (**b**) structures pre- and post- feedback. *Dashed lines* and *boxes* indicate new concepts and linking statements

As seen in the 1st draft CM of student 2 (see Fig. 3a), similar to the 1st draft of student 1, the CM represented a *Spoke* structure, consistent with superficial knowledge of the ICF and health conditions in the case study. The 1st CM is so elementary that any additions to the central concepts would not have disrupted the existing knowledge structure. In addition there is no integration between the patient’s personal and clinical presentation and the appropriate component of the ICF. In the 2nd, post-feedback CM (see Fig. 3b), the student was able to build on their basic knowledge of the first map, incorporating new concepts in a meaningful way by using appropriate explanatory phrases (concepts in dashed lines and associated linking statements). For example, the student not only linked the predisposing factors of TB to the contextual component of the ICF, but also more specifically to the personal factors. Furthermore, the student demonstrated their understanding of the interplay between the components of the functioning domain of the ICF by linking the patient-specific symptoms to have an influence on the activity component. Based on Hay’s criteria [15] for demonstrating meaningful learning, it is evident that the second map is a significant improvement over the first.

**Fig. 4:**
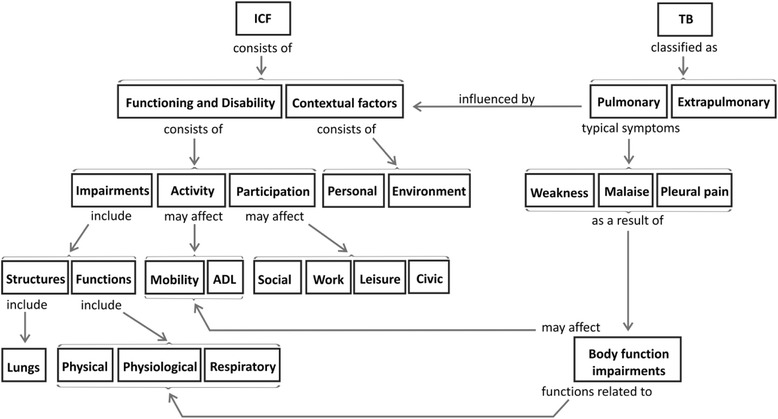
Student 3 first draft map with *Net* structure

In the excerpt taken from the 1st draft (pre-feedback) of student 3 (see Fig. 4 below), the *Net* structure demonstrating the interrelationship between concepts is evident. Underlying a more complex mental model is the assumption that concepts relate to each other and that there are multiple possibilities of creating meaningful linkages. The student was able to unpack the ICF not only in terms of its main components but was also able to recognize the interplay between the components of the functioning domain. Furthermore, the student was able to link most of the manifestations of TB to the appropriate functioning component of the ICF.

## Discussion

The results of the study support our hypothesis that structured feedback improved the graphical complexity of students’ declarative models of two important knowledge domains. Furthermore, the enhanced complexity of most final CM’s - albeit at different levels of understanding - provides additional evidence for incorporating structured feedback in a meaningful way based on the premise that learning is an active process of knowledge construction.

In this study, Kinchin’s criteria were used for evaluating the graphical presentation of CM’s. Crudely, the results revealed that the majority of CMs, especially the ones initially classified as *Spoke* or *Chain*, became more complex. The *Spoke-* and *Chain-*structured maps, on second evaluation, advanced at least one step in complexity, evident in significantly fewer *Spoke* structures and significantly more *Net* structures in the final evaluation. The pretest-posttest design of the task allowed for the evaluation of personal changes in knowledge and how temporality, i.e. the use of invalid links earlier in the knowledge structure to support valid relationships later on, was dealt with in order to promote a sound knowledge framework [8, 14, 15].

The conceptual basis of Kinchin’s criteria is thought to represent patterns of learning, i.e. meaningful (deep)-, rote-, and non-learning [15, 25]. In our data, some initial *Spoke* and *Chain* structures did not demonstrate change over time. For the *Spoke* structures, five out of eighteen CM’s remained unchanged, indicating that erroneous statements were not corrected, valid additions were not made, and that opportunities for development (benchmarking) were left unutilized. Similarly, 22 out of the 33 *Chain* structures remained unchanged. Although *Spoke* and *Chain* structures are indicative of ‘learning readiness’ and ‘active and flexible’ learning respectively, it appeared that some students failed to integrate structured feedback, and lacked the ability to deconstruct/reconstruct their naive ideas concerning these knowledge domains. What we do not know from our data are the reasons why suggestions for further exploration of concepts were not incorporated by these students. Perhaps the feedback itself may have lacked clarity, or the time period between receiving feedback and submitting the final CM was insufficient for students to resolve all gaps in their knowledge. In order to enhance the personal learning and developmental process of students, an educator-student interview might be one approach to understand the misconceptions and gaps in knowledge that prevent students from advancing to richer knowledge frameworks [1, 11].

Most of the literature using concept mapping attempts to unpack one knowledge domain, whereas this study sought to evaluate the integration of two medically-related knowledge domains, i.e. the understanding of the consequences of a health condition (TB) using the ICF as a conceptual model. Although students are expected to apply the ICF in the clinical context, the understanding of health conditions within an operative conceptual model is often taught in a decontextualised manner. This was our first attempt to contextualise the manifested experience of a health condition - using a case-based approach - within the desired model. A previous study found similar effects of better integration of concepts when using a problem-based learning environment [26, 27]. Using CM’s within the classroom environment could be valuable to determine the level of understanding prior to exposing students to the clinical context, and provides the opportunity to remediate misconceptions and gaps in their knowledge.

This study has some limitations that need consideration for future work. Although quasi-experimental designs are often used in the education research field, the results from these studies should be taken with caution due to threats to the internal validity and lack of control group. Another limitation relates to whether the structured feedback or engagement with learning resources mediated the improvements noted in the structural and conceptual configuration of CMs. Learning is an active and dynamic process and results concerning the impact of educator’s feedback should be interpreted with caution until we better understand how feedback disrupts students’ initial understanding of concept(s) and what strategies they adopt to rectify gaps in knowledge. In order to establish the true effect of structured, formative feedback, future research should include a control group but also identify factors related to improvements following feedback. We also do not know how much ‘time-on-task’ was spent on the first draft submission. Evidence exists to support the notion that ‘time-on-task’ is a proxy for learning and achievement [28]. Therefore, we cannot rule out the possibility that students spent limited time on the draft and more on the final submission. Future research could position this methodology within Kirkpatrick’s model to better understand reactions to the implementation of a new ‘intervention’ but also to assess changes in behavior in the clinical context.

## Conclusion

This study provides preliminary evidence of the effect of structured, formative feedback on students’ improved understanding of important topics in physiotherapy undergraduate education. Giving feedback on CM’s provides educators with insight into students’ personal difficulties in the learning process. This could then be used to implement strategies for addressing misconceptions in essential conceptual relationships between different knowledge domains, prior to students entering the clinical context.

## Abbreviations

CM: Concept mapping
ICF: International Classification of Functioning, Disability and Health
PABAK-OS: Prevalence Adjusted Bias Adjusted Kappa- Ordinal Scale
TB: Tuberculosis

## References

[1] Laurillard D (2002). Rethinking university teaching: a conversational framework for the effective use of learning technologies.

[2] Schmidmaier R, Eiber S, Ebersbach R, Schiller M, Hege I, Holzer M (2013). Learning the facts in medical school is not enough: which factors predict successful application of procedural knowledge in a laboratory setting?. BMC Med Educ..

[3] Seely Brown J, Collins A, Duguid P (1989). Situated cognition and the culture of learning. Educ Res.

[4] Baroody AJ, Feil Y, Johnson AR (2007). An alternative reconceptualization of procedural and conceptual knowledge. J Res Math Educ.

[5] Kumar S, Dee F, Kumar R, Velan G (2011). Benefits of testable concept maps for learning about pathogenesis of disease. Teach Learn Med.

[6] Torre DM, Daley B, Stark-Schweitzer T, Siddartha S, Petkova J, Ziebert M (2007). A qualitative evaluation of medical student learning with concept maps. Med Teach.

[7] Alonso-Tapia J (2002). Knowledge assessment and conceptual understanding. Reconsidering conceptual change: issues in theory and practice.

[8] Novak JD (1998). Learning, creating and using knowledge: concept maps as facilitative tools in schools and corporations.

[9] Novak JD, Godwin DB (1984). Learning how to learn.

[10] Burgess A, Mellis C (2015). Feedback and assessment for clinical placements: achieving the right balance. Adv Med Educ Pract.

[11] Evans C (2013). Making sense of assessment feedback in higher education. Rev Educ Res.

[12] Hay DB, Wells H, Kinchin IM (2008). Quantitative and qualitative measures of students learning at university level. High Educ.

[13] Kinchin IM (2001). Can a novice be viewed as an expert upside-down?. Sch Sci Rev.

[14] Kinchin IM, Hay DB, Adams A (2000). How a qualitative approach to concept map analysis can be used to aid learning by illustrating patterns of conceptual development. Educ Res.

[15] Hay DB (2007). Using concept maps to measure deep, surface and non-learning outcomes. Stud High Educ.

[16] WHO (2001). ICF, towards a common language for functioning, disability and health.

[17] Novak JD, Cañas A J. The theory underlying concept maps and how to construct and use them. Florida: Institute for Human and Machine Cognition; 2008. Available at: http://cmap.ihmc.us/Publications/.

[18] Ghosh S (2007). Combination of didactic lectures and case-oriented problem-solving tutorials toward better learning: perceptions of students from a conventional medical curriculum. Adv Physiol Educ.

[19] Boud D, Molloy E (2013). Feedback in higher and professional education: understanding it and doing it well.

[20] Price M, Handley K, Millar J, O’Donovan B (2010). Feedback: all that effort but what is the effect?. Assess Eval High Educ.

[21] Ausubel DP (1963). The psychology of meaningful verbal learning.

[22] Sim J, Wright CC (2005). The kappa statistic in reliability studies: use, interpretation, and sample size requirements. Phys Ther.

[23] Landis JR, Koch GG (1977). The measurement of observer agreement for categorical data. Biometrics.

[24] Ausubel DP (1977). The facilitation of meaningful verbal learning in the classroom 1. Educ Psychol.

[25] Jarvis P (1992). Paradoxes of learning.

[26] Hung C-H, Lin C-Y (2015). Using concept mapping to evalute knowledge structure in problem-based learning. BMC Med Educ.

[27] Dyer J-O, Hudon A, Montpetit-Tourangeau K, Charlin B, Mamede S, van Gog T (2015). Example-based learning: comparing the effects of additionally providing three different integrative learning activities on physiotherapy intervention knowledge. BMC Med Educ.

[28] Admiraal W, Wubbels T, Pilot A (1999). College teaching in legal education: teaching method, students’ time-on-task, and achievement. Res High Educ.

